# Changes in HIV treatment differentiated care uptake during the COVID‐19 pandemic in Zambia: interrupted time series analysis

**DOI:** 10.1002/jia2.25808

**Published:** 2021-10-28

**Authors:** Youngji Jo, Sydney Rosen, Karla Therese L. Sy, Bevis Phiri, Amy N. Huber, Muya Mwansa, Hilda Shakwelele, Prudence Haimbe, Mpande M. Mwenechanya, Priscilla Lumano‐Mulenga, Brooke E. Nichols

**Affiliations:** ^1^ Section of Infectious Diseases Department of Medicine Boston Medical Center Boston Massachusetts USA; ^2^ Department of Global Health Boston University School of Public Health Boston Massachusetts USA; ^3^ Health Economics and Epidemiology Research Office Department of Internal Medicine School of Clinical Medicine Faculty of Health Sciences University of the Witwatersrand Johannesburg South Africa; ^4^ Department of Epidemiology Boston University School of Public Health Boston Massachusetts USA; ^5^ Clinton Health Access Initiative Lusaka Zambia; ^6^ Ministry of Health Lusaka Zambia; ^7^ The Centre for Infectious Disease Research in Zambia Lusaka Zambia; ^8^ Department of Medical Microbiology Amsterdam University Medical Centre Amsterdam The Netherlands

**Keywords:** antiretroviral treatment, COVID‐19, differentiated service delivery, HIV service delivery, multi‐month dispensing, Zambia

## Abstract

**Introduction:**

Differentiated service delivery (DSD) models aim to improve the access of human immunodeficiency virus treatment on clients and reduce requirements for facility visits by extending dispensing intervals. With the advent of the COVID‐19 pandemic, minimising client contact with healthcare facilities and other clients, while maintaining treatment continuity and avoiding loss to care, has become more urgent, resulting in efforts to increase DSD uptake. We assessed the extent to which DSD coverage and antiretroviral treatment (ART) dispensing intervals have changed during the COVID‐19 pandemic in Zambia.

**Methods:**

We used client data from Zambia's electronic medical record system (SmartCare) for 737 health facilities, representing about three‐fourths of all ART clients nationally. We compared the numbers and proportional distributions of clients enrolled in DSD models in the 6 months before and 6 months after the first case of COVID‐19 was diagnosed in Zambia in March 2020. Segmented linear regression was used to determine whether the outbreak of COVID‐19 in Zambia further accelerated the increase in DSD scale‐up.

**Results and discussion:**

Between September 2019 and August 2020, 181,317 clients aged 15 or older (81,520 and 99,797 from 1 September 2019 to 1 March 2020 and from 1 March to 31 August 2020, respectively) enrolled in DSD models in Zambia. Overall participation in all DSD models increased over the study period, but uptake varied by model. The rate of acceleration increased in the second period for home ART delivery (152%), ≤2‐month fast‐track (143%) and 3‐month MMD (139%). There was a significant reduction in the enrolment rates for 4‐ to 6‐month fast‐track (−28%) and “other“ models (−19%).

**Conclusions:**

Participation in DSD models for stable ART clients in Zambia increased after the advent of COVID‐19, but dispensing intervals diminished. Eliminating obstacles to longer dispensing intervals, including those related to supply chain management, should be prioritized to achieve the expected benefits of DSD models and minimize COVID‐19 risk.

## 1 INTRODUCTION

In 2020, an estimated 16.4 million people living with human immunodeficiency virus (PLHIV) and taking antiretroviral treatment (ART) in sub‐Saharan Africa risked treatment interruptions because of COVID‐19 due to closing or limiting of human immunodeficiency virus (HIV) services, antiretroviral supply chain disruptions, transportation or travel restrictions and/or overwhelmed service providers [1]. Maintenance of ART services — in addition to continued case identification and prompt initiation of newly diagnosed PLHIV on lifelong treatment — is critical to protect the progress that has been made towards HIV epidemic control [2].

One potential solution to the disruptions caused by COVID‐19 is differentiated service delivery (DSD), a “client‐centered approach that simplifies and adapts HIV services across the cascade to serve the needs of PLHIV better and reduce unnecessary burdens on the health system” [3]. DSD has emerged as a key strategy for HIV programmes in resource‐limited settings, as DSD models can lessen the burden of HIV treatment on clients and providers by extending medication dispensing intervals, reducing requirements for facility visits and adjusting the location of service delivery [4]. These adjustments also minimise client contact with healthcare facilities and other clients [5], a high priority during the COVID‐19 pandemic.

In Zambia, the Ministry of Health began promoting DSD models for ART in 2016, with participation gradually increasing over time [6]. By February 2021, roughly a quarter of the country's nearly 1 million clients were recorded as having ever been enrolled in a DSD model [7]. The models offered in Zambia included multi‐month dispensing (MMD), fast‐track medication pickup, community adherence groups (CAGs) and home ART delivery, with healthcare facilities varying widely on which of these or other models they adopted (Table 1). Three‐month dispensing has been the standard of care for stable clients [8], though it has not been universally implemented. The Ministry of Health introduced 6‐month dispensing in 2019 [9]. When the country's first SARS‐CoV‐2 infection was confirmed in March 2020, the Ministry of Health doubled down on the implementation of 6‐month dispensing for all patients from ART initiation, with the exception of 3‐month dispensing for those aged 2–10 [10]. Other models became more or less attractive in the face of COVID‐19 risks and restrictions, depending on whether they required clients to meet as groups (e.g. CAGs) or reduced the need for public interaction (e.g. home delivery). In this study, we assessed the association between the COVID‐19 pandemic and Zambia's response to it and the rate of change of enrolment in DSD models in the 6‐month period before and after diagnosis of the first SARS‐CoV‐2 case.

**Table 1:** Description of each of the evaluated differentiated service delivery models implemented in Zambia between September 2019and August 2020^a^

Differentiated service delivery model	Description
Fast‐track (≤2 months, 3 months, 4–6 months)	A model that creates a separate queue, kiosk, or procedure at a facility to speed up service delivery for stable clients [5]. In Zambia, this typically involves a separate and shorter queue for quick dispensing when a clinical visit is not indicated.
Multi‐month dispensing (MMD) (3 months, 4–6 months)	Any model in which the primary goal is to dispense medications for a longer duration than is done under standard care (usually 3 or 6 months) [5]. Dispensing is typically done alongside a clinical facility‐based visit.
Community adherence group (CAG)	Group of ±6 people, based on residential proximity or client preference, meet monthly at a designated place in the community. Members collect medication at clinical appointments for other CAG members, in a rotating fashion [4].
Home ART delivery	Trained community health workers (CHWs) linked to facilities conduct home visits to deliver ART, conduct health screening, monitor adherence and refer clients as required. All community services are captured on a tablet‐based SmartCare linked Community HTC (HIV testing and counseling) or Community ART module [4].
Others	There are a number of additional models currently enrolling clients in Zambia, but all at a relatively small scale. These models include: ART dispensing after/before (standard clinic) hours, weekend clinic, scholar (i.e. expanded hours, focused on school‐going youth), central dispensing unit, community ART distribution points/pharmacy, health post, mobile ART distribution (in hard‐to‐reach areas) and rural/urban adherence groups (i.e. pre‐packed ART dispensed by a healthcare worker in a group setting outside of typical clinic hours).

^a^Eligibility for all models was identical – “stable” adult clients (except for the scholar model, which was aimed at school‐going adolescents). Eligibility did not change as a response to the pandemic.

ART, antiretroviral therapy.

## 2 METHODS

To assess how DSD model enrolment, by model type, changed before and after the start of the COVID‐19 pandemic, we conducted a retrospective review of SmartCare, Zambia's national electronic medical record system. As of February 2021, 737,411 clients were recorded in SmartCare as currently on ART, representing roughly three quarters of all ART clients in the country. The remaining quarter of clients attend facilities that do not yet utilize SmartCare. We accessed records for all clients aged 15 or older who newly enrolled in any DSD model between September 2019 and August 2020 at any of 737 health facilities across all 10 provinces. Children younger than the age of 15 were not included in the study protocol, given that when the protocol was written, children were not eligible for DSD models. We collapsed the many DSD models recorded in SmartCare into eight groups based on the location and duration of medication dispensing: ≤2‐month fast‐track, 3‐month fast‐track, 4‐ to 6‐month fast‐track, 3‐month MMD, 4‐ to 6‐month MMD, CAGs, home ART delivery and all others. A description of each model can be found in Table 1 [11].

We first describe the basic characteristics of clients enrolled by DSD model before and after the introduction of COVID‐19 in Zambia to determine whether enrolment in models has changed in terms of location (urban/rural, level of health facility) or in the age or sex distribution of clients enrolling. For each of the DSD model groups, we calculated the number of DSD enrolments by month from September 2019 to August 2020. To assess the effect of the COVID‐19 pandemic on DSD care utilisation, we conducted an interrupted time series analysis using a segmented regression. Segmented regression has been previously used to evaluate changes at any defined point in time [12]. In our analysis, we compared the change in slope between the cumulative number of clients enrolled in DSD before 1 March 2020, compared to 1 March through August 2020 (i.e. before and after 1 March 2020), the approximate date when COVID‐19 was first diagnosed in Zambia [13]. We used the following segmented regression model: $DSD_{t}=\beta_{0}\;+\beta_{1}time+\;\beta_{2}covid_{t}+\;\;\beta_{3}time\cdot covid_{t}$, where *time* is in months, and *covid* is a dummy variable indicating whether the current time is pre‐ or post‐COVID. The outcome *DSD* is the cumulative number of clients enrolled in DSD at time *t*. $\beta_{3}$ indicates the slope change following the intervention, which we then tested whether there was a significant change in $\beta_{3}$ before and after 1 March 2020; a significant change in slope would suggest that DSD utilisation changed substantially during the COVID‐19 pandemic. All analyses were performed at a two‐sided significance level of 0.05. Finally, we estimated percentage changes in participation between the periods for each model group based on the mean slope. Data analysis was conducted in R version 4.0.2. (The R Project for Statistical Computing, Vienna, Austria.)

### 2.1 Ethics

This study protocol was approved by ERES Converge IRB (Zambia), protocol number 2019‐Sep‐030; the Human Research Ethics Committee (Medical) of the University of Witwatersrand, protocol number M190453; and the Boston University IRB H‐38823 for the use of data with a waiver of consent.

## 3 RESULTS AND DISCUSSION

Participation in DSD models before and after the introduction of COVID‐19 in March 2020 is presented in Table 2. Between September 2019 and August 2020, 181,317 clients aged 15 or older were recorded as being newly enrolled in DSD models in Zambia in the SmartCare electronic medical record system. These include 81,520 before and 99,797 on or after 1 March 2020, an overall increase of 22.4%. However, uptake varied widely by model. For example, the number of clients most substantially increased for home ART delivery (168%), 3‐month MMD (96%), ≤2‐month fast track dispensing (69%) but decreased for 4‐ to 6‐month fast‐track dispensing (−26%) and other models (−20%). Between the two periods, 3‐month dispensing increased from 13% to 21% of all DSD enrolments, ≤2‐month fast‐track from 7% to 10% and home ART delivery from 1% to 2%. While 4‐ to 6‐month fast‐track declined, 4‐ to 6‐month MMD increased between the two time periods, due to the greater increase in DSD enrolment in rural areas where fast‐track is seldom implemented.

**Table 2:** Percentage change in numbers of clients enrolled in DSD models before and after COVID‐19 introduction in Zambia (*n* = 181,317)

Parameters	Number of clients (proportion change %)	Location		Healthcare level			Sex			
		Urban	Rural	Health post	Clinic	Hospital	Male	Female		
All models	Before^a^	81,520 (100%)	64,997	16,523	4587	49,619	27,314	28,562	46,808	42 (11)
	After^b^	99,797 (100%)	75,424	24,373	5218	58,403	36,176	36,386	59,587	41 (12)
	%Δ^c^	22%	16%	48%	14%	18%	32%	27%	27%	–
≤2‐month fast‐track dispensing	Before	6005 (7%)	4405	1600	302	4202	1501	2078	3927	40 (12)
	After	10,163 (10%)	7205	2958	618	6854	2691	3654	6509	39 (11)
	%Δ	69%	64%	85%	105%	63%	79%	76%	66%	–
3‐month fast‐track dispensing	Before	6325 (8%)	5788	537	318	3973	2034	2067	4258	41 (10)
	After	6917 (7%)	6140	777	350	4360	2207	2427	4490	41 (11)
	%Δ	9%	6%	45%	10%	10%	9%	17%	5%	–
4‐ to 6‐month fast‐track dispensing	Before	19,112 (23%)	18,283	829	2013	10,026	7073	6481	12,631	43 (10)
	After	14,168 (14%)	13,627	541	975	7553	5640	5172	8996	43 (10)
	%Δ	−26%	−25%	−35%	−52%	−25%	−20%	−20%	−29%	–
3‐month MMD	Before	10,743 (13%)	8215	2528	410	7101	3232	3744	6999	41 (11)
	After	21,101 (21%)	14,812	6289	1122	13,030	6949	7564	13,537	41 (12)
	%Δ	96%	80%	149%	174%	83%	115%	102%	93%	–
4‐ to 6‐month MMD	Before	30,832 (38%)	22,447	8385	998	19,689	10,145	11,246	19,586	44 (11)
	After	38,120 (38%)	28,260	9860	1439	21,576	15,105	14,172	23,948	43 (11)
	%Δ	24%	26%	18%	44%	10%	49%	26%	22%	–
Community adherence groups	Before	2885 (4%)	1595	1290	112	1628	1145	917	1968	45 (11)
	After	3483 (3%)	1362	2121	133	2220	1130	1231	2252	45 (11)
	%Δ	21%	−15%	64%	19%	36%	−1%	34%	14%	–
Home ART delivery	Before	721 (1%)	444	277	240	132	349	283	438	39 (12)
	After	1929 (2%)	1472	457	288	838	803	686	1243	39 (12)
	%Δ	168%	232%	65%	20%	535%	130%	142%	184%	–
Others	Before		3820							40 (13)
	After	3916 (4%)	2546	1370	293	1972	1651	1480	2436	39 (13)
	%Δ	−20%	−33%	27%	51%	−31%	−10%	−15%	−23%	–

^a^ Before: September 2019 to February 2020.

^b^ After: March 2020 to August 2020.

^c^ Percentage change in participant numbers between before and after periods.

DSD, differentiated service delivery; MMD, multi‐month dispensing.

The proportion of all DSD enrolments in 4‐ to 6‐month fast‐track fell from 23% to 14%. There was no change in the proportions of clients enrolled in 4‐ to 6‐month MMD (38% of all DSD enrolments in both periods). Participation of clients enrolled in rural areas increased for ≤3‐month fast‐track, 3‐month MMD, CAGs and others. Home ART delivery was the only model to see a relative increase in the proportion of clients enrolled in urban areas (Table 2). There were no significant differences between the two time periods in the composition of the population enrolled in terms of sex or age.

**Figure 1:**
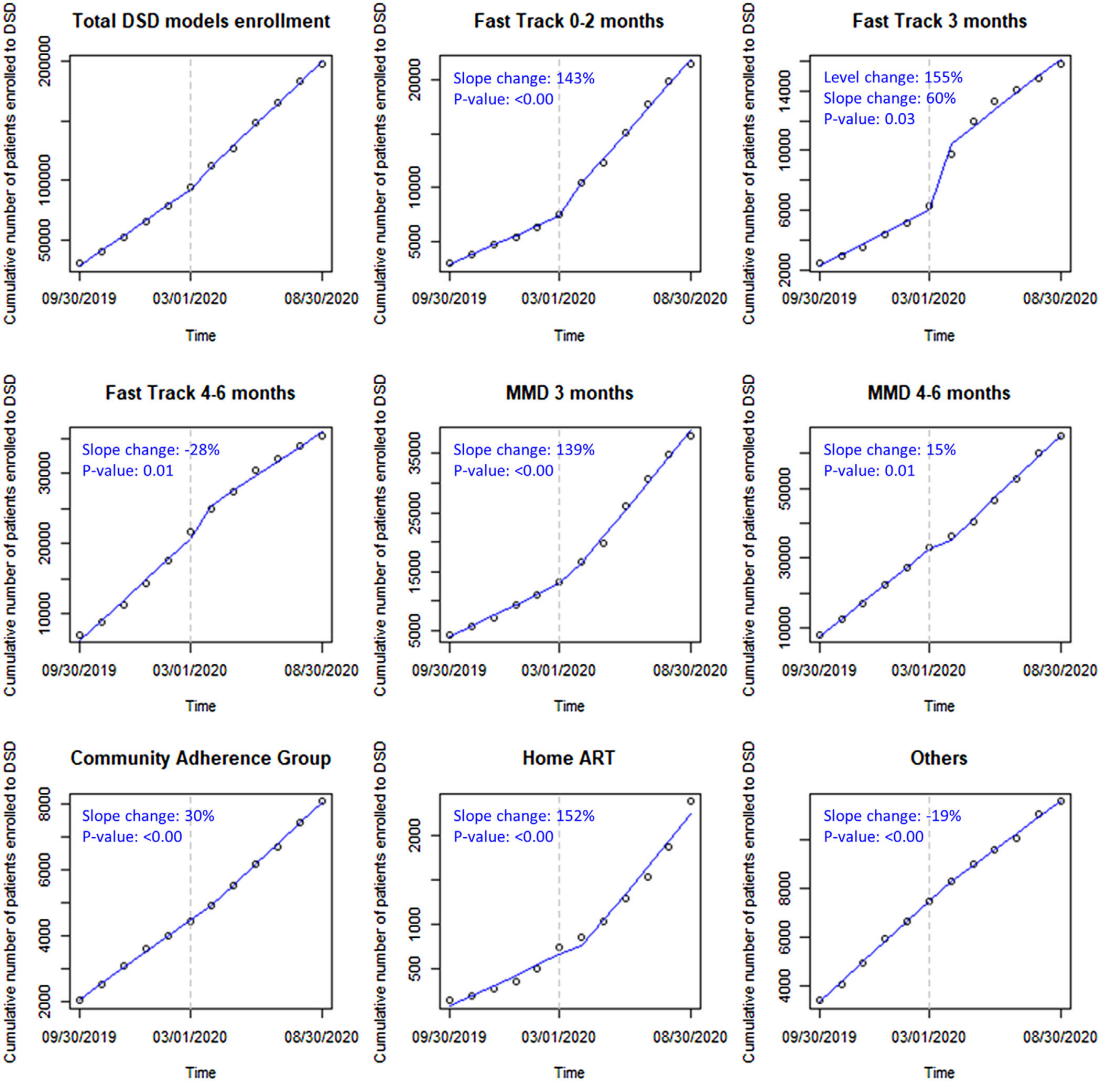
Interrupted time series scatter plot and slope lines for the DSD models before (September 2019 to February 2020) and after (March 2020 to August 2020) 1 March 2020 in Zambia. ART, antiretroviral therapy; DSD, differentiated service delivery; MMD, multi‐month dispensing.

Participation in DSD models accelerated over the study period. Comparing the periods before and after 1 March 2020, segmented linear regression models demonstrated an acceleration in the rate of increase (significant increases in slope) in participation during the COVID‐19 pandemic for home ART delivery (152% change in slope between periods, *p*‐value <0.001), ≤2‐month fast‐track (143%, *p* < 0.001) and 3‐month MMD (139%, *p* < 0.001). Three‐month fast‐track showed both an immediate increase in numbers enrolled (155% from 6278 to 9729) and a significant acceleration in the rate of increase (60%, *p* = 0.03) between the two periods. In contrast, there were significant decelerations in the increase in enrolment for 4‐ to 6‐month fast‐track (−28%, *p* = 0.01) and for “other” models (−19%, *p* < 0.001) (Figure 1).

Over the course of 2020, the COVID‐19 pandemic was associated with accelerated participation in DSD models in Zambia, though with uneven increases across the models. Most new clients enrolled in ≤2‐month fast track, 3‐month MMD, 4‐ to 6‐month MMD, CAGs or home ART delivery. On the other hand, the increase in DSD enrolment was slower for the 4‐ to 6‐month fast‐track and “other” models. Participation in home ART delivery increased the most (168%), but it still accounted for only a small proportion of all participation (2%). Recommendations that high‐risk individuals remain at home, to minimise their exposure to SARS‐CoV‐2, may potentially explain the expansion of home delivery models. We also found an immediate jump in enrolment for ≤2‐month and 3‐month fast‐track on 1 March 2020 and an increase 1 month later for home ART delivery.

Although 3‐ to 6‐month dispensing is Zambia's national policy for stable patients, the proportion of clients newly enrolled in 4‐ to 6‐month DSD models fell between the two time periods, while ≤3‐month dispensing increased for new DSD model enrolees. Another study at the United States President's Emergency Plan For AIDS Relief (PEPFAR)‐supported sites found that 6‐month dispensing had been expanded to 56% of clients (*n* = 561,409) by July 2020, an increase from fewer than 50,000 in September 2019 in Zambia [7]. PEPFAR global data, excluding South Africa, showed a similar trend across its global programmes with an increase in 3‐ to 6‐month dispensing from 46% in December 2019 to 69% by the end of June 2020 [14]. The smaller relative increase in 4‐ to 6‐month dispensing in this analysis compared to the general nationwide (e.g. not DSD enrolee specific) dispensing data for Zambia, as well as global PEPFAR data, is likely due to the fact that we focused solely on patients newly enrolling into a DSD model (i.e. their first interaction with a DSD model only). This analysis is thus not reflective of the total scope of 4‐ to 6‐month dispensing during the COVID‐19 pandemic, but of new DSD enrolees alone. It is possible that new DSD enrolees receive ≤3‐month dispensing at first, but then switched to 4‐ to 6‐month dispensing during the pandemic period [15].

Our study has several limitations. We relied entirely on routinely collected medical record data from the SmartCare system, which covers only about three quarters of Zambia's ART facilities. It is possible that healthcare facilities without SmartCare differ from those in our data set in ways that would affect our outcomes. For example, facilities without SmartCare may be more poorly resourced or more remotely located than those with SmartCare, characteristics that could lead to differential uptake of DSD models. While interrupted time series analysis allows the ability to control for secular trends in the data (unlike pre/post cross‐sectional studies) using population‐level data with clear graphical presentation of results, this analysis does not illustrate how and why the introduction of COVID‐19 resulted in different scale‐up patterns by DSD models and whether and to what extent the temporal changes may differ by setting. Future research may examine the drivers and barriers of MMD from both the demand and supply‐side aspects in the context of COVID‐19 to improve continuation of care. Moreover, we have not considered retention in and switching between the DSD models or care more generally. Future work should aim to understand how this rapid acceleration of DSD model uptake has affected overall initiation and retention in care from a longitudinal cohort population perspective.

## 4 CONCLUSIONS

Based on national electronic medical record data for clients enrolled in DSD models in Zambia from September 2019 to August 2020, our findings suggest that the introduction of the COVID‐19 pandemic was associated with an acceleration in the scale‐up of DSD models for clients on ART in Zambia. Efforts to eliminate obstacles to longer dispensing intervals should be prioritised to achieve the expected benefits of DSD models and minimise COVID‐19 risk. This process has already begun in Zambia, where the government is now recommending relaxation of eligibility criteria for MMD, such that all clients initiating ART to receive a 3‐month or 6‐month supply of medications immediately, allowing them to delay their first follow‐up visit for 3 months or 6 months after initiation [16]. Evaluating the impact of this evolution in DSD guidelines will be a high priority for the coming years.

## COMPETING INTERESTS

The authors declare that they have no conflicting interests.

## AUTHORS' CONTRIBUTIONS

YJ, SR, and BEN conceived the study. YJ, SR, KTLS and BEN designed the study. BP, MM, HS, PH, MMM and PLM led study data collection. YJ, KTLS analysed the data and SR, ANH, BEN contributed to data analysis. YJ, SR, KTLS and BEN wrote the first draft of the manuscript. All authors reviewed and edited the manuscript. All authors have read and approved the final manuscript.

## FUNDING

Funding for the study was provided by the Bill & Melinda Gates Foundation through OPP1192640 to Boston University. Youngji Jo was supported by the Ruth L. Kirschstein National Research Service Award, National Institutes of Health T32 Training Grant (grant number: T32 AI052074‐14).
